# Not Just Another Headache: Cerebral Venous Sinus Thrombosis in a Patient With Isolated Antithrombin III Deficiency

**DOI:** 10.7759/cureus.8383

**Published:** 2020-05-31

**Authors:** Dawood Findakly, Jack Komro

**Affiliations:** 1 Internal Medicine, Creighton University Arizona Health Education Alliance/Valleywise Health Medical Center, Phoenix, USA; 2 Internal Medicine, Kirksville College of Osteopathic Medicine, A. T. Still University, Kirksville, USA

**Keywords:** cerebral venous sinus thrombosis (cvst), papilledema, antithrombin iii, anticoagulation, mr venography, hematology laboratory

## Abstract

Cerebral venous sinus thrombosis (CVST) is a rare condition. Symptoms and signs arise from a combination of thrombosis of cerebral veins and increased intracranial pressure. The most common presenting symptom is a non-descriptive headache, but presentation varies with underlying etiology. CVST requires a high index of suspicion to diagnose, particularly in those without apparent risk factors. Evaluation and diagnosis should include a combination of a thorough history, ophthalmoscopic examination, laboratory studies, and imaging. Management is multidimensional and aims to reverse the underlying causes, and prompt treatment with anticoagulation by heparin to decrease thrombotic burden, risk of permanent neurological deficits, and death. In the present study, we report the case of a 61-year-old man referred to the emergency department by an ophthalmologist for bilateral papilledema and eventually diagnosed with CVST secondary to isolated antithrombin III deficiency. Although CVST is uncommon, this case is worthwhile to report as the presentation is unique, and it requires a high index of clinical awareness for timely diagnosis and early therapeutic intervention.

## Introduction

Cerebral venous sinus thrombosis (CVST) is a rare condition that occurs in up to four adults per million per year, has a slightly higher incidence in children aged less than 18 years, and is more common in females given gender-specific situations [1-3]. The pathophysiology includes thrombosis of cerebral veins impairing venous drainage, increasing intracranial pressure, and disrupting CSF absorption [4]. Etiologies responsible for the above mechanisms include thrombophilia, chronic inflammatory conditions, female gender-specific risks, malignancy, infection, medications, and trauma [4-6]. The presentation varies with the underlying etiology, chronicity, and location of thrombosis. The most common presenting symptom, however, is an uncharacteristic headache which may mimic many other conditions [5,7,8]. In a systematic review, papilledema was found in more than 80% of patients in which direct ophthalmoscopic examination was performed [9]. Basic laboratory studies, including coagulation studies and screening for prothrombotic conditions, are recommended in the initial workup [7]. Although not the imaging modality of choice, non-contrast computed tomography (CT) of the head is often done early in the evaluation and is helpful to rule out certain causes of CVST [4]. Magnetic resonance (MR) and MR with venography (MRV) are the diagnostic tests of choice when CVST is suspected because of accessibility and non-invasive technique compared to the gold standard, digital subtraction angiography (DSA) [7]. The goals of management include implementing a multidisciplinary approach to reduce thrombosis load by first treating underlying reversible causes [4]. Heparin is the recommended first-line treatment and may be required lifelong if CVST was provoked by an underlying prothrombotic state [7]. The following is a case of an adult male diagnosed with CVST on brain imaging after presenting with no known risk factors, non-specific symptoms, and ophthalmoscopic findings concerning for increased intracranial pressure. Appropriate diagnosis is essential to provide proper treatment and improve outcomes.

## Case presentation

**Figure 1:**
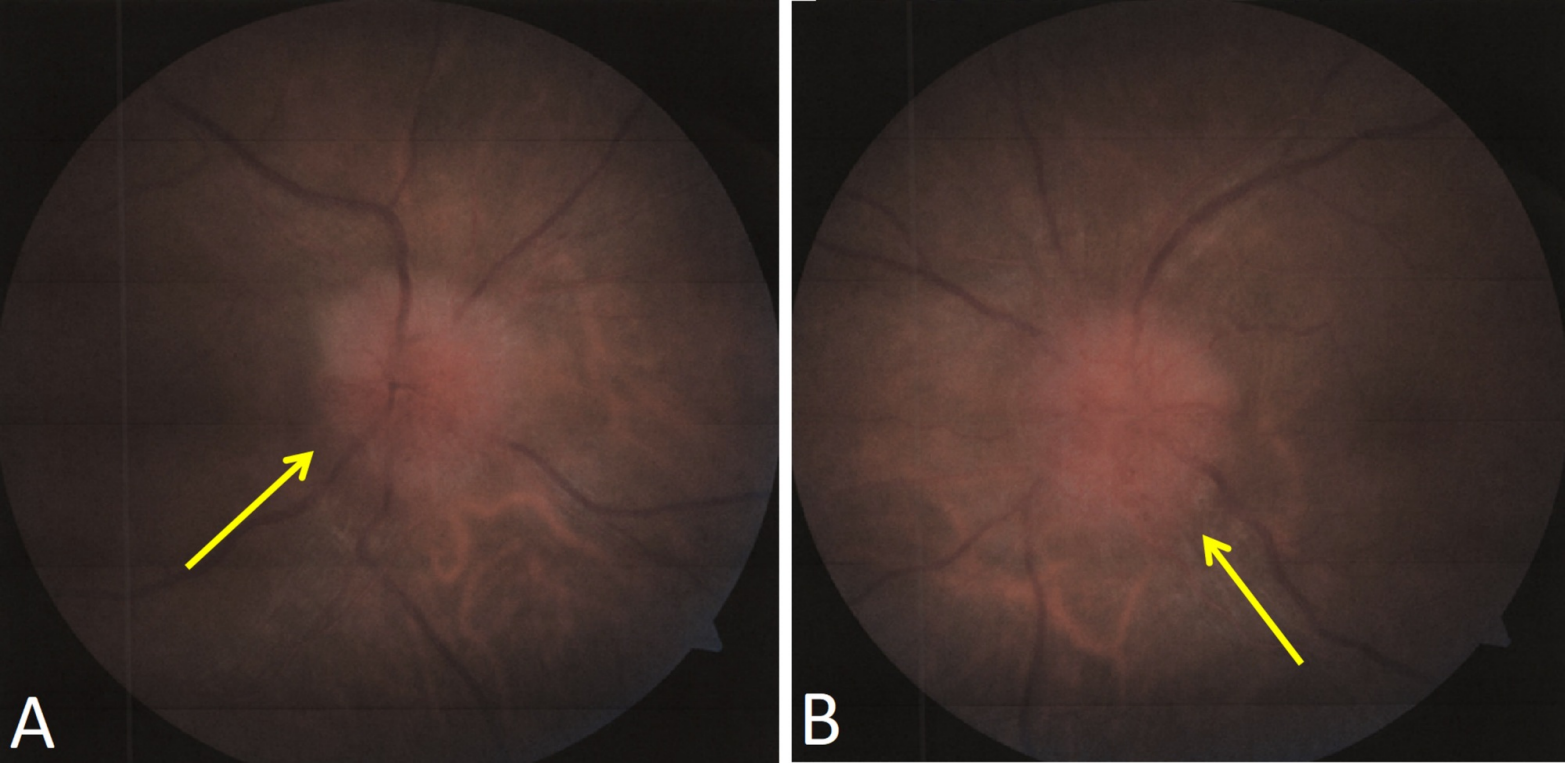
Direct ophthalmoscope examination of the patient’s optic nerve representing bilateral papilledema. The yellow arrows denote swollen optic nerves in each eye. (A) right optic disc, (B) left optic disc

A 61-year-old Hispanic man with a past medical history of benign essential hypertension, pre-diabetes, and class 1 obesity (body mass index 30.5 kg/m^2^) was referred from an ophthalmology clinic to the emergency department for findings consistent with bilateral papilledema and suspicion for raised intracranial pressure (Figure 1). 

The patient reported blurry vision and worsening severe headache for a few days before his presentation. On examination, the patient was vitally stable except for an elevated blood pressure of 163/90 mmHg. He was alert and oriented, with no motor or sensory deficits. Initial laboratory blood test results and renal and liver function tests were unremarkable, and a sickle cell screen was negative. Coagulation workup was normal, except for a low antithrombin III (ATIII) level at 71% (Table 1).

**Table 1:** Summary of the patient's laboratory and procedure results WBC: white blood cell; Hgb: hemoglobin; PLT: platelet; ALP: alkaline phosphatase; AST: aspartate aminotransferase; ALT: alanine aminotransferase; PT: prothrombin time; INR: international normalized ratio; PTT: partial thromboplastin time; APLS: antiphospholipid syndrome; ANA: antinuclear antibodies; APC-R: activated protein C resistance; FVL: factor V Leiden; AT III: antithrombin III; Ag: antigen; HIV: human immunodeficiency virus; CSF: cerebrospinal fluid; RBC: red blood cell; GS: gram stain; Cx: culture; PMNL: polymorphonuclear leukocyte

Test	Normal reference range and units	Patient's results upon presentation
WBC	3.4-11.0 × 10^3/µL	8.2 × 10^3/µL
Hgb	11.3-16.8 g/dL	15.6 g/dL
PLT	147-395 K/µL	292 K/µL
Sodium	137-145 mmol/L	141 mmol/L
Potassium	3.5-5.1 mmol/L	4.8 mmol/L
Chloride	98-107 mmol/L	105 mmol/L
Bicarbonate	22.0-30.0 mmol/L	26 mmol/L
Creatinine	0.66-1.25 mg/dL	1.29 mg/dL
Calcium	8.4-10.2 mg/dL	9.7 mg/dL
Albumin	3.2-4.6 g/dL	4.8 g/dL
Magnesium	1.6-2.3 mg/dL	2.2 mg/dL
Total bilirubin	0.2-1.3 mg/dL	0.5 mg/dL
ALP	56-119 U/L	109 U/L
AST	17-59 U/L	32 U/L
ALT	4-50 U/L	39 U/L
PT	9.8-12.2 seconds	11.1 seconds
INR	0.9-1.1	1.0
PTT	27.7-38.3 seconds	28.7 seconds
APLS workup	Negative	Negative
ANA titer IgG	None detected	None detected
Factor V activity	62%-140%	84%
APC-R ratio	>=2.00	5.23
FVL mutation	Negative	Negative
AT III	82%-136%	71%
Coccidioides Ag and antibody testing	Negative	Negative
HIV	Non-reactive	Non-reactive
CSF glucose	40-70 mg/dL	53 mg/dL
CSF protein	12-60 mg/dL	50 mg/dL
CSF total nucleated cells	0-10 mm^3^	0 mm^3^
CSF RBC	<=0 mm^3^	0 mm^3^
CSF GS, Cx	No growth	No PMNL, no organisms detected
CSF fluid coccidioides Ag	Negative	Negative

**Figure 2:**
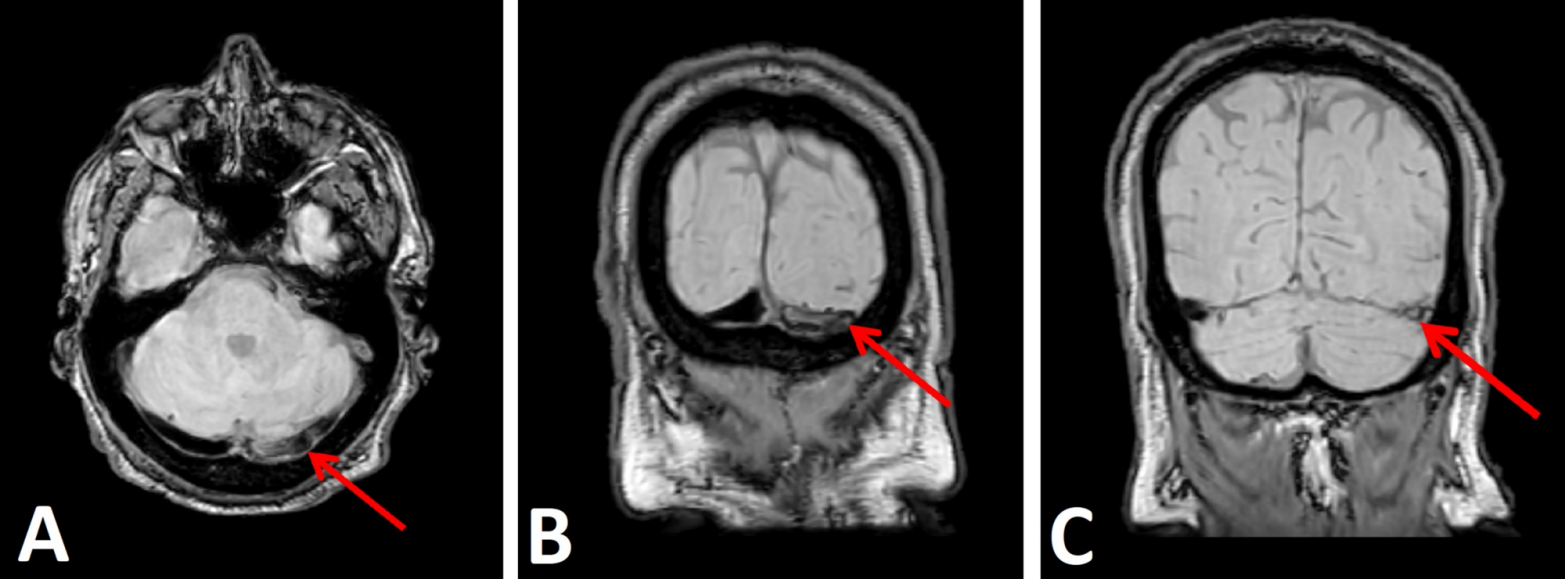
Magnetic resonance venogram: (A) axial view showing a left transverse sinus thrombosis (arrow); (B) coronal view showing a left transverse sinus thrombosis (arrow); (C) coronal view showing a left sigmoid sinus thrombosis (arrow)

Lumbar puncture was also performed, and cerebrospinal fluid testing ruled out meningitis. The patient was monitored in the intensive care unit, where he was treated with anticoagulation and underwent an unenhanced CT of the head, which was negative for acute intracranial abnormality. Subsequent contrast‑enhanced brain MRI with MRV and CT venogram (CTV) with IV contrast revealed findings consistent with chronic non-occlusive thrombosis of the left transverse sinus, left sigmoid sinus, and left internal jugular vein at the left internal jugular vein fossa and canal in the posterior skull with extensive collateralization in the head and neck (Figure 2). The patient was treated and discharged home in stable condition.

## Discussion

CVST is uncommon, represents less than 1% of all strokes, and is often overlooked due to a non-characteristic headache frequently being the primary symptom at presentation [1,2,7]. Therefore, the clinician must have a high suspicion for diagnosis if a patient presents with a headache and has one or more known risk factors for CVST [4,10]. This case illustrates how in those without typical or previously identified risk factors, a comprehensive investigation through a detailed history, physical examination including ophthalmoscopic examination, and pertinent laboratory studies under most recent guidelines are warranted [4,9,10].

A number of systematic reviews have described the characteristics of CVST, including its presenting symptoms, risk factors, imaging modalities, and patient outcomes. An overview of recent systematic reviews is useful to further explain the uniqueness of the present case. The presentation of CVST is highly variable; however, it commonly presents with at least one of a few symptoms. The most common presenting symptoms include headache, focal neurological deficits, and seizures [4,5,9]. Headache is by far the most common, with 70% to 90% of patients presenting with this symptom [4,9,11]. Papilledema is also a common finding, which represents elevated intracranial pressure. Although papilledema is not required to diagnose CVST, it can be a critical finding that would further raise suspicion for the diagnosis. Despite this, it has been inconsistently reported in patients diagnosed with CVST. One systematic review found that only 12 out of 30 case reports commented on papilledema and that papilledema was present in 10 out of those 12 cases [9]. Risk factors for CVST have also been described extensively, but may not be present in up to 15% of cases [4]. Common risk factors include prescription drug use, thrombophilia, prior infection, head trauma, cancer, and chronic inflammatory conditions [5,9,11]. A significant number of women manifest with gender-specific risk factors, such as pregnancy, puerperium, oral contraceptive pill use, or hormone replacement therapy [4,9,11].

Another challenge to diagnosing CVST is the use of proper imaging to visualize the defect. Head CT is often the initial imaging used, although it is only positive in about 30% of the CVST cases [4,9,12]. CTV has significantly improved sensitivity compared to other CT imaging techniques [4,11]. The imaging test of choice is MRV, and the combination of MRV and MRI provides the most sensitive imaging for diagnosing CVST as they provide the best view of the cerebral venous systems [4,7]. Although DSA is considered the gold standard for diagnosing CVST, it is typically only utilized if CTV or MRV is inconclusive [4,11,13]. Other imaging studies less commonly used that perhaps need more research into their utility for CVST include direct venous venography, positron emission tomography (PET), and CT perfusion scan [4]. Once the diagnosis of CVST is made, the outcome is favorable, and most patients live, although women have more favorable outcomes than men [1,8,9,14]. Regardless, the potential for permanent neurological deficits and death makes it a diagnosis that should not be missed.

To our knowledge, the present case is unique because it is only one of a few reports of an adult with CVST due to isolated AT III deficiency [15,16]. The patient was 61 years old and male, both of which are unordinary, as most patients are less than 50 years old and female [1,4,9]. Moreover, the patient had no known pre-existing risk factors at presentation. Despite males appearing to have poorer outcomes than females, this patient was eventually discharged in a stable condition without neurological deficits. Perhaps the higher frequency of unfavorable outcomes is because of the lack of reasonable consideration for CVST in males compared to females, leading to delayed diagnosis and opportunity for complications to occur. This case suggests that outcomes may be more favorable with a high index of suspicion and a complete workup, including comprehensive coagulation tests and imaging.

## Conclusions

Given the infrequency of reported CVST cases and its non-specific presenting symptoms, more cases that describe risk factors and patients' presentations are necessary to identify patterns that promote early recognition of CVST. Timely recognition and efficient workup, along with prompt treatment with anticoagulation, can prevent potentially avoidable consequences, including death.
